# Determinants of herbivory on seedlings in a subtropical forest: Insights from plant apparency, Janzen-Connell effects, and functional traits

**DOI:** 10.1016/j.pld.2026.03.009

**Published:** 2026-03-17

**Authors:** Gang Zhou, Daniel F. Petticord, Yao-Zhan Xu, Yuan-Zhi Qin, Biao Dong, Xiu-Juan Qiao, Ming-Xi Jiang

**Affiliations:** a State Key Laboratory of Plant Diversity and Specialty Crops, Wuhan Botanical Garden, Chinese Academy of Sciences, Wuhan 430074, China; b Cornell University, Ithaca 14850, United States; c University of Chinese Academy of Sciences, Beijing 100871, China; d College of Horticulture and Gardening, Yangtze University, JingZhou 434025, China; e School of Life Sciences, Central China Normal University, Wuhan 430079, China

**Keywords:** Ant-plant mutualisms, Forest dynamic plot, Plant apparency, Plant functional traits, Seedling herbivory

## Abstract

Herbivory is widely known to play a critical role in structuring plant communities. However, much less is known about what factors determine herbivory in forest ecosystems. Here, we monitored herbivory damage on more than 2000 seedlings in a subtropical forest (2022 and 2023), to identify the key biotic drivers of interspecific variation in seedling herbivory. We found a positive correlation between seedling height and herbivory, evidence for the “plant apparency” hypothesis. Both increasing proportion of herbivory on local conspecifics and conspecific adult density were associated with higher herbivory on focal seedlings, evidence for the Janzen-Connell hypothesis. Interestingly, interactions with ants, as an example of interspecific mutualisms, were found to increase herbivory damage, while decrease seedling mortality in the same research system, highlighting the pivotal role of biotic mutualisms in mediating plant–herbivore interactions. Furthermore, leaf functional traits accounted for the greatest proportion of variance in seedling herbivory, indicating that they served as the primary determinant regulating herbivory pressure at the seedling stage. Overall, our study provides insight into the intricate interplay of seedling height, Janzen-Connell effect and functional traits in determining seedling herbivory and consequently assembly of forest ecosystems. These findings advance our understanding of subtropical forest community dynamics and provide empirical support for evidence-based conservation strategies in such ecosystems.

## Highlights

• Seedling height exhibited a positive effect on seedling herbivory, evidence for the “plant apparency” hypothesis.
• Plant chemical content and the synthesis of secondary metabolites could strongly influence standing herbivory.
• Higher density of heterospecific ant-associated seedlings generally reduced standing herbivory on focal seedlings.

## 1 Introduction

Herbivory exerts a profound influence on the growth, survival, and reproduction of plants, and thus obviously is pivotal in shaping plant communities (Crawley, 1989; Burkepile and Parker, 2017). Approximately 20% of net annual primary production in terrestrial ecosystems is consumed by herbivores (Cyr and Face, 1993). Importantly, the selective pressure exerted by herbivory varies across species within plant communities (Agrawal and Weber, 2015), meaning herbivory may ultimately influence community dynamics.

Several hypotheses have been put forward to explain why certain plants are preferred by herbivores over others. Some of these hypotheses are straightforward, such as the apparency hypothesis, which states that plants more easily detected by herbivores are more likely to be consumed (Feeny, 1976; Stamp, 2003). The plant apparency hypothesis has been used to illustrate why certain climax species - those ‘bound to be found’ (Feeny, 1976) - tend to invest more in chemical compounds for defense against herbivore damage and get support in subtropical forest (Yuan and Wang, 2025). However, this contrasts with more cryptic species which can avoid predator detection outright (Grossman et al., 2019; Kurokawa et al., 2022; Zhang et al., 2023).

The Janzen-Connell (J-C) hypothesis is arguably the most prevalent framework used to describe how interactions between herbivores and plants may ultimately structure plant communities. In brief, the J-C hypothesis proposes a form of conspecific negative density dependence (CNDD), wherein host-specific predators/pathogens disproportionately attack conspecific individuals in proximity to parent trees and consequently diversity emerges because heterospecific may escape this intense pressure (Janzen, 1970; Connell, 1971). A related concept, the herd protection hypothesis, suggests that high heterospecific plant density acts as a buffer against herbivores, reducing interactions between host plants and their natural enemies (Wills and Green, 1995; Peters, 2003). Similarly, the associational resistance hypothesis suggests that individuals with a higher diversity of neighboring species generally exhibit improved resistance to herbivory (Atsatt and O'Dowd, 1976; Barbosa et al., 2009). These hypotheses (e.g., Janzen-Connell, associational resistance) generally suggest that seedling herbivory may increase with the density of conspecific individuals and decrease with increasing density and diversity of heterospecific neighbors, though this pattern is context-dependent. In contrast, alternative hypotheses—including the resource specialization hypothesis and more individuals hypothesis—and empirical findings (Yuan and Wang, 2025) highlight more complex relationships: higher heterospecific diversity can sometimes increase herbivory by providing a broader resource base for generalist herbivores, or have neutral effects depending on community composition and herbivore functional traits.

This idea that herbivory may promote diversity by pushing certain plants to specialize along certain trait axes has merit (i.e., cryptic and defenseless v. apparent and chemically defended) (Sentis et al., 2020). A fundamental growth-defense trade-off has been proposed as an extension of this: slow-growing species prioritize constitutive defenses (e.g., lignin, phenolic compounds) to resist herbivory broadly over the course of a long lifetime. Conversely, fast-growing species tend to rely more heavily on short-term induced defenses, risking fitness via quicker growth against reduced capacity to sustain prolonged herbivory (Coley et al., 1985). This concept is interwoven with consumer-resource dynamics; nutrient limitation may push plants towards slower-growing, intensively defensive strategies, whereas nutrient surplus allows for riskier acquisitive strategists to dominate. Evidence that plants assemble in ‘niches’ along this growth-defense axes is widespread (Giolai and Laine, 2024).

Moreover, we have become increasingly aware of the role of other species in moderating herbivory. For example, it has been established that mutualisms between trees and other taxa can protect against herbivores much the same way that a suite of secondary chemical metabolites or other defensive investments might (Massad et al., 2011). Perhaps the most well-documented example of these mutualisms is that observed between plants and ants (Janzen, 1966; Heil and Kost, 2006; Clark and Singer, 2018). Recent research suggested that ant as a buffer could mitigate Janzen-Connell effect of plants with extrafloral nectaries (EFNs) (Zhou et al., 2024a). In addition, topography—via metrics including slope, aspect, and elevation—modulates seedling herbivory by shaping microclimatic conditions (e.g., temperature, humidity) and resource availability (e.g., soil moisture, nutrient distribution), which in turn influence herbivore foraging behavior and plant defensive trait expression (Alexandre et al., 2018; Kozlov et al., 2022). For example, steeper slopes reduce humidity and herbivore activity, while convex vs. concave terrain alters soil nutrient accumulation, indirectly affecting herbivory via plant nutrient content (Alexandre et al., 2018). Although numerous hypotheses have been put forward to explain the variations in herbivory among plants, it remains unclear which hypotheses are relatively important in subtropical forests and whether mutualistic interactions can mediate the herbivory process.

**Fig. 1:**
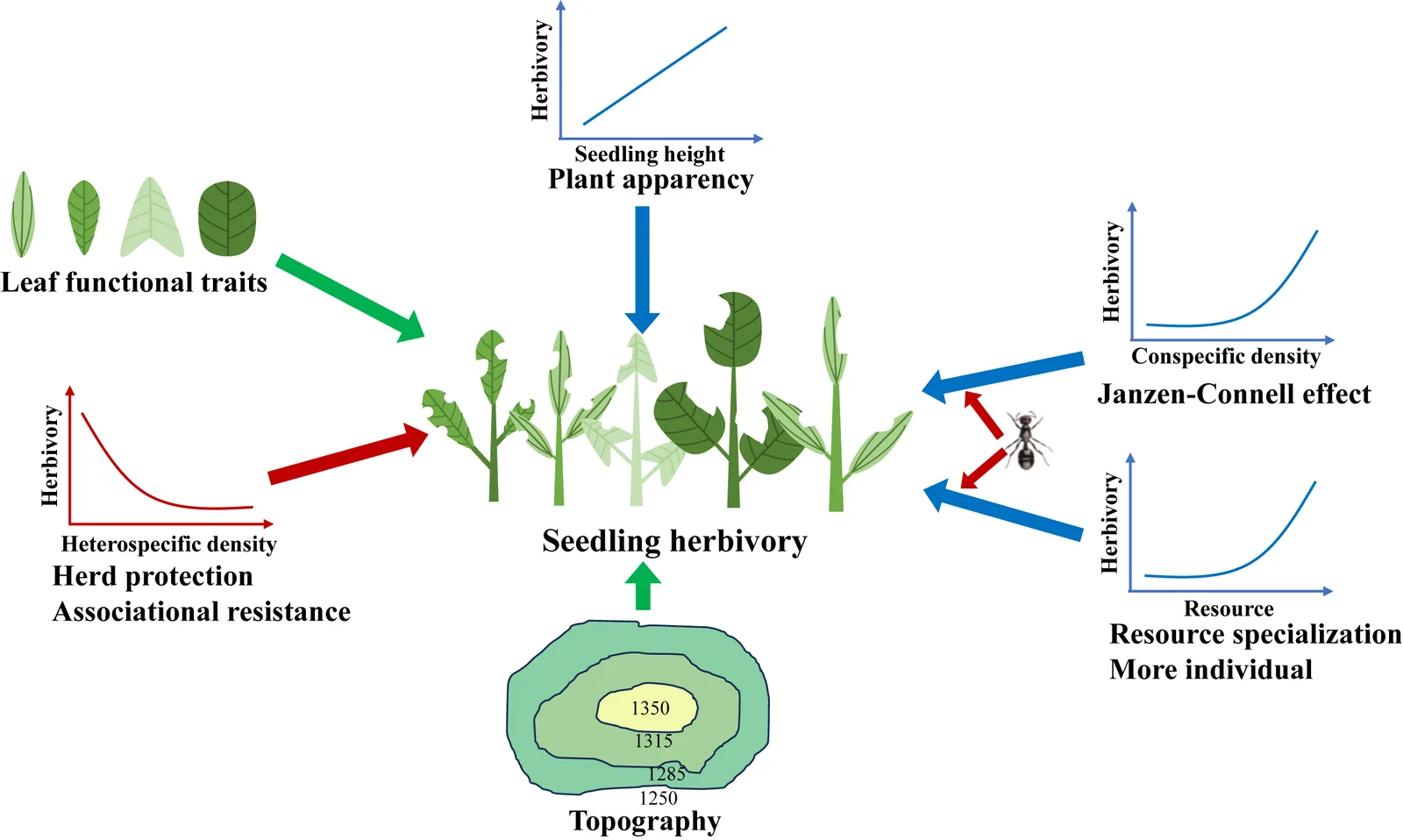
Conceptual diagram showing the potential effects of each hypothesis on seedling herbivory and the potential effects of ants on herbivory behavior of herbivores. A standardized color scheme is adopted for the arrows: blue for facilitating effects, red for mitigating effects, and green for unknown effects. The plant apparency hypothesis posits that plants that are more conspicuous or easily visible are prone to higher rates of herbivore attack compared to cryptic species. In contrast, the Janzen-Connell hypothesis suggests that the seedlings located near adult individuals of the same species are more susceptible to herbivory due to host-specific predator or pest pressure. Additionally, the herd protection and associational resistance hypotheses propose that seedlings surrounded by a greater number of heterospecific neighbors are less likely to be exposed to herbivores, leading to enhanced resistance to herbivory. However, the resource specialization hypothesis and more individual hypothesis show that seedling with higher neighbors' diversity will provide more resource for herbivores and subsequently suffer more herbivory. We utilized ‘leaf damage’, characterized by small bite marks on leaves, as an indicator of herbivory risk.

In this observational study, we investigated the incidence of seedling herbivory within a 25-ha subtropical forest dynamic plot in Badagongshan, central China. Our objective was to elucidate the extent to which plant apparency, Janzen-Connell effects, and ant mutualisms, as well as other herbivory-related hypotheses, jointly determine levels of seedling herbivory. We expected to see a direct correlation between the level of herbivory on seedlings and both seedling height (which we interpret as a measure of plant apparency) and the density of conspecific individuals (the J-C and herd protection hypotheses). Likewise, we expected a negative relationship between herbivory and both the density and diversity of nearby heterospecific individuals (Fig. 1). Building on increasing interest in how interspecific mutualisms generate positive density-dependent effects that counteract Janzen-Connell dynamics—and on our prior finding that ant-plant mutualisms mitigate these effects (Zhou et al., 2024a)—we hypothesized that ant-hosting plants would be less sensitive to biotic neighborhood influences on herbivory. Specifically, we predicted that ant presence would buffer against herbivory driven by conspecific density, and local seedling diversity (Fig. 1).

## 2 Materials and methods

### 2.1 Study site

This study was conducted within the Badagongshan (BDGS) forest dynamics plot (FDP), a mixed evergreen and deciduous broad-leaved mixed forest located in Hunan province in Central China (coordinates: 29°46ʹN, 110°05ʹE). The BDGS FDP is part of an international collaborative project focused on documenting forest dynamics across the globe (Condit, 1998). The region experiences a subtropical climate characterized by warm temperatures averaging 11.5 °C annually (range: 0.1–23.3 °C) and abundant precipitation (∼2105 mm annually). Since 2011, comprehensive monitoring has been conducted within the BDGS FDP, including identification, tagging, diameter at breast height (DBH) measurement, and spatial mapping of all trees ≥ 1 cm DBH at five-year intervals (Zhang et al., 2020). In 2012, a seed rain census was established within the BDGS FDP, utilizing 135 seed traps (each 0.5 m^2^) and 405 seedling plots (each 1 m^2^), organized into census stations (one seed trap and three seedling plots per station). Between 2012 and 2024, censuses occurred twice annually (May and August), totaling 24 surveys. This biodiversity and detailed monitoring framework provide a context for studying biotic neighborhood influences and mutualistic interactions affecting seedling herbivory.

### 2.2 Standing leaf herbivory

Herbivory was assessed on 2123 and 2052 seedlings observed, with DBH < 1 cm, during July 2022 and July 2023 censuses, respectively. Herbivory rate was assessed by scoring leaf damage for each seedling following a 6-tier ordinal scale (scores 1–6) with objective criteria: 1 = no damage; 2 = 1–20% leaf area damaged; 3 = 20–40% leaf area damaged; 4 = 40–60% leaf area damaged; 5 = 60–80% leaf area damaged; 6 = 80–100% leaf area damaged. If leaf count exceeded 100 visually, herbivory was estimated from three branches facing different cardinal directions (Wang et al., 2023), consistently assessed by the same observer to minimize bias:

$$H\mathrm{e}rb\text{ivory}\;\text{Rate}\;\left(\text{HR}\right)=\sum_{i=1}^{n}\left(C_{i}\times M_{i}\right)/C$$

where C_i_ is the leaf count at each loss level, M_i_ is the median leaf-loss proportion at each (*i*th) level, and C is the total leaf count per seedling.

### 2.3 Leaf functional traits and analysis

Leaf functional traits were measured from ten healthy, fully expanded leaves per seedling collected in June 2022 and each species was selected ten individuals (Tables S1 and S2). To avoid disturbing the seedling inside the seedling plot, we selected the individuals located adjacent to seedling plot for sampling. For individuals with fewer than 10 leaves, all leaves were collected after removing those with severe damage.

Physical traits included leaf thickness, leaf area (LA), specific leaf area (SLA, leaf area/dry mass), and specific leaf weight (SLW, dry mass/leaf area). Specifically, the ten healthy, fully expanded leaves were scanned by CANON LIDE-220 and then calculated the leaf area by leaf analysis software of WANSHEN (www.wseen.com). Subsequently, the ten leaves were dried for 48 h in oven with 72 °C then weighed by electronic balance with an accuracy of 0.1 mg.

The leaf chemical elements including carbon (C), nitrogen (N), phosphorus (P), kalium (K), calcium (Ca), sodium (Na), magnesium (Mg), iron (Fe), aluminum (Al), cuprum (Cu) and manganese (Mn). And then, chemometrics can be calculated which including leaf carbon-to-nitrogen ratio (C:N), leaf carbon-to-phosphorus ratio (C:P) and leaf nitrogen-to-phosphorus ratio (N:P). The C and N determined by Elementar (FXL 950, America). Other elements determined by Optical Emission Spectrometer (OPTIMA 8000DV, America). The specific analytical methods are described in the appendix.

Due to trait autocorrelation (Fig. S1), principal component analyses (PCA) were performed separately on elemental composition and organic compounds. PCA for chemical elements and chemometrics identified two components: PC1 driven by total N and total P; and PC2 driven by calcium (Ca) and carbon (C) content (Fig. S2). Additionally, PC1 explained 32.6% of variance, and PC2 explained 17.2%. The leaf organic matters including flavonoid, soluble protein, tannin, fructose, alkaloid and chlorophyll. The second PCA we generated based on organic compound content revealed PC1 was primarily driven by proteins and tannins and PC2 by alkaloids (Fig. S3). Additionally, PC1 explained 39.9% of variance, and PC2 explained 19.0%. The details of measurement could find in appendix.

### 2.4 Biotic neighborhood

We assessed plant density distributions using a multi-scale spatial framework: Conspecific (*D*_*con*_) and heterospecific (*D*_*het*_) adult tree densities were calculated as inverse-distance weighted basal areas within a 20-m radius:

$$D=\sum_{i=1}^{n}\frac{b_{i}}{d_{i}}$$

where b_i_ is basal area (cm^2^), d_i_ is the distance in meters from each *i*th individual to the center of the seedling trap, and *n* is the number of individuals (conspecific or heterospecific).

Seedling density was defined as the number of seedlings per 1-m^2^ plot, distinguishing conspecific and heterospecific individuals relative to the focal seedling. Ant-seedling interaction strength (*E*_*i*_) was calculated (Song et al., 2023; Zhou et al., 2024b):

$$E_{i}=\frac{r_{i}-n_{i}}{r_{i}+n_{i}}$$

where r_i_ is the proportion of ant co-occurrence with tree species *i*, and *n* is species' *i*'s relative abundance among seedlings. Positive *E*_*i*_ values indicate ant preference; negative *E*_*i*_ values indicate ants avoid these plants (Table S2). Specifically, E_i_ represents the intensity of the associative relationship between seedlings and ants, and its quantification simultaneously considers the abundance of both seedlings and ants—this design enables E_i_ to reflect the actual ecological coupling degree between the two groups in the study system (i.e., higher E_i_ values indicate a closer association between seedlings and ants, while lower values indicate a weaker association). The ant-seedling interaction strength (*E*_*i*_) was derived from co-occurrence data collected during the seedling re-census for all seedling plots in May and August of 2021 and 2022.

Based on *E*_*i*_, the heterospecific seedling density were categorized into seedlings preferred by ants and seedlings avoided by ants. In addition, the diversity of ant-preferred seedlings and ant-avoided seedlings were estimated separately.

### 2.5 Topography and seedling height

Variation in topography around the site was incorporated into the model following methods from Lu et al. (2015), which quantified mean elevation, slope gradient, and topographic aspect within the BDGS FDP study area. Seedling height, served as a proxy for plant apparency, was measured at each census following Martini et al. (2021).

### 2.6 Nearby density of herbivory events

We investigated whether the observed herbivory on focal seedlings could be explained by the density of herbivory events on neighboring conspecific or heterospecific seedlings. Specifically, “Conspecific Herbivory Density” is a plot-level metric designed to quantify the local herbivory pressure exerted by herbivores associated with conspecific (same-species) seedlings on a focal seedling, independent of the herbivory damage directly observed on the focal seedling itself, calculated as the sum of herbivory rate on neighboring conspecific seedlings in the seedling plot. Similarly, heterospecific herbivory density was quantified as the total herbivory rate on neighboring heterospecific seedlings in the same seedling plot. The calculation formula of conspecific and heterospecific herbivory density followed as:

$${HD}_{con}=\frac{\sum_{i=1}^{n}{HR}_{con,i}}{A}$$

$${HD}_{het}=\frac{\sum_{j=1}^{m}{HR}_{het,j}}{A}$$

n: Number of conspecific neighbors within the sampling plot (1m × 1m) around the focal seedling; m: Number of heterospecific neighbors within the same sampling plot; HR_con,i_: The herbivory rate of conspecific neighbors; HR_het,j_: The herbivory rate of heterospecific neighbors; A: Area of the sampling plot (1 m^2^).

### 2.7 Statistical analyses

We employed variation partitioning to quantify the relative importance of each group for herbivory rate (Legendre, 2008). We conducted the full model to estimate the contributions of variation of herbivory rate for each group. For each group, we summed the estimates and then divided the total estimates of the full model to get the relative contributions. The groups of predictors included: seedling height, leaf functional traits, herbivory density, biotic environment and topography. Leaf functional traits encompass leaf thickness, SLA, SLW, and LA, chemical elements (PC1 chemical element and PC2 chemical element), and organic content (PC1 organic compounds and PC2 organic compounds). Herbivory density includes conspecific herbivory density and heterospecific herbivory density. Biotic environments include the interaction strength between ants and plants (E_i_), conspecific adult density, heterospecific adult density, conspecific seedling density, heterospecific ant-preferred seedling density, heterospecific ant-avoided seedling density, ant-preferred seedling richness, and ant-avoided seedling richness. Topography includes slope, mean elevation, and aspect. Prior to model fitting, all continuous variables were standardized using the scale function (Becker et al., 1988) to ensure comparability of variable importance (Gelman and Hill, 2006), and collinearity was controlled via variance inflation factor (VIF) checks (threshold: VIF < 10; Zuur et al., 2010).

Before conducting structural equation model (SEM), we aggregated the group that contains many factors into one factor firstly. For each group, we employed multiple regression models to estimate the impact of leaf functional traits, biotic environment, topographical characteristics, and the density of herbivory events on herbivory rate. The variables of each group same as the variation partitioning models. We used the ‘*performance*’ package in R to check the collinearity of variables for each multiple regression model (Lüdecke et al., 2021).

We used Piecewise SEM (Lefcheck, 2016) to further examine predictor associations with herbivory, incorporating species and seedling plot as random effects. Groups with multiple variables were aggregated using multiple regressions prior to structural equation modeling (SEM). We constructed the conceptual diagram of SEM to predict the associations between herbivory and each group (Fig. S4). For example, the topography could influence the leaf functional traits, herbivory density or biotic environment and then impact the seedling herbivory. To validate the robustness of these predictor–response relationships, the piecewiseSEM method accounted for random effects of tree species and seedling plot, providing ‘marginal’ (the variation explained by fixed effect) and ‘conditional’ (the variation explained by fixed and random effect) contributions of predictors to herbivory dynamics. Model fits were evaluated by Fisher's C-test (acceptable range: 0.05 < *P* < 1.00). Analyses were performed in R v.4.2.2 (R Core Team, 2020) using the “*lme4*” (Bates et al., 2015) and “*piecewiseSEM*” (Lefcheck, 2016) packages.

## 3 Results

A total of 50,346 leaves from 2123 seedlings in 2022 and 50,306 leaves from 2052 seedlings in 2023 were examined (for detailed species-level data, refer to Appendix S1: Table S2). The weighted average herbivory rate was 9.91% (SE = 0.74%, *N* = 51 species) in 2022 and 9.50% (SE = 0.73%, *N* = 51 species) in 2023 when including all individuals of 51 species.

### 3.1 The relative importance of each group for variation of herbivory rate

**Fig. 2:**
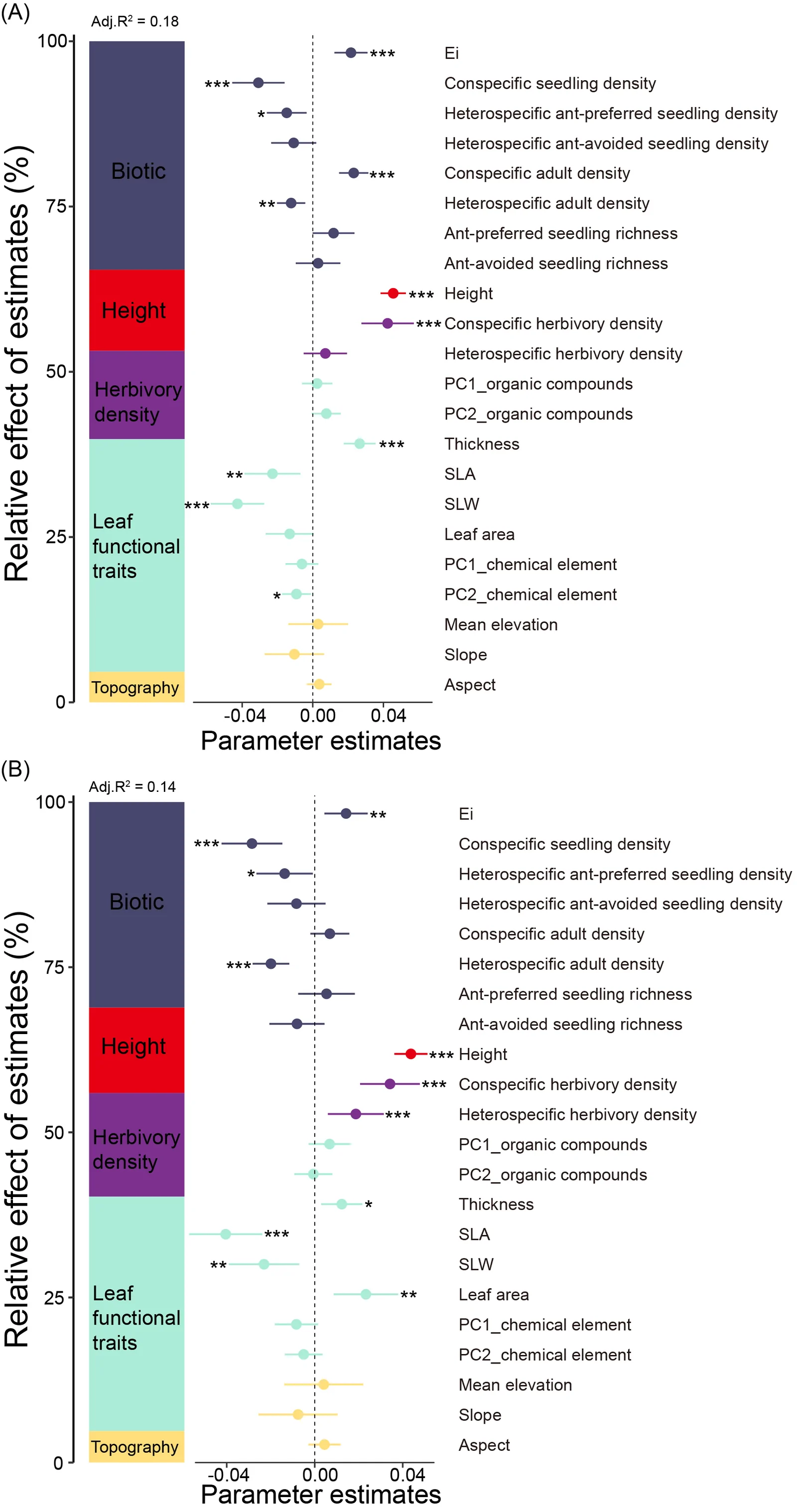
Drivers of herbivory rate. Biotic and abiotic predicters of herbivory rate in 2022 (A, *n* = 2123) and 2023 (B, *n* = 2052). Multiple regression shows the relative importance of predicters of herbivory rate with 95% confidence intervals, ∗*P* < 0.05, ∗∗*P* < 0.01, ∗∗∗*P* < 0.001. The Ei represents the strength of ant-plant interaction. The SLA is specific leaf area and the SLW is specific leaf weight. Bar graphs illustrate the importance of each group of predictors, represented as the proportion of explained variance.

The full model incorporating five variable groups accounted for up to 18% and 14% (adjusted R^2^, Fig. 2A and B) of the total observed variation in herbivory rate in 2022 and 2023. Among these five groups of explanatory variables, leaf functional traits were the most important, and accounted for 35.2% and 35.5% of explainable variation in herbivory in 2022 and 2023, respectively (equivalent to approximately 5–6% of the total variation in herbivory) (Fig. 2A and B). Among leaf functional traits, leaf physical properties, such as SLA and SLW, were the main contributing factors (Fig. 2A and B).

The second most important category of factors was the influence of the biotic environment. In 2022, the biotic neighborhood could account for 34.5% of the total explanatory power among the five groups, and in 2023, this proportion was 31.1%. Notably, even after controlling seedling height, herbivory density, leaf functional traits and topography, a positive association between *Ei* (ant-plant association) and herbivory rate persisted (Fig. 2A and B). Seedling density and heterospecific adult density were each associated with reduced herbivory rate. We found seedling richness had no effect on herbivory rate (Fig. 2A and B).

The third most important group of factors was the density of herbivory events, which could explain 13.3% of the variation in focal seedling herbivory in 2022 and 15.6% of that variation in 2023 among the five groups. Focal seedling herbivory increased with rising density of local herbivory events (Fig. 2A and B). Seedling height, our proxy for ‘apparency’ accounted for 12.3% and 12.9% in 2022 and 2023, respectively (Fig. 2A and B). Topography was the least effective explanatory framework, and had no significant impact on herbivory rate.

### 3.2 The direct and indirect divers of seedling herbivory

**Fig. 3:**
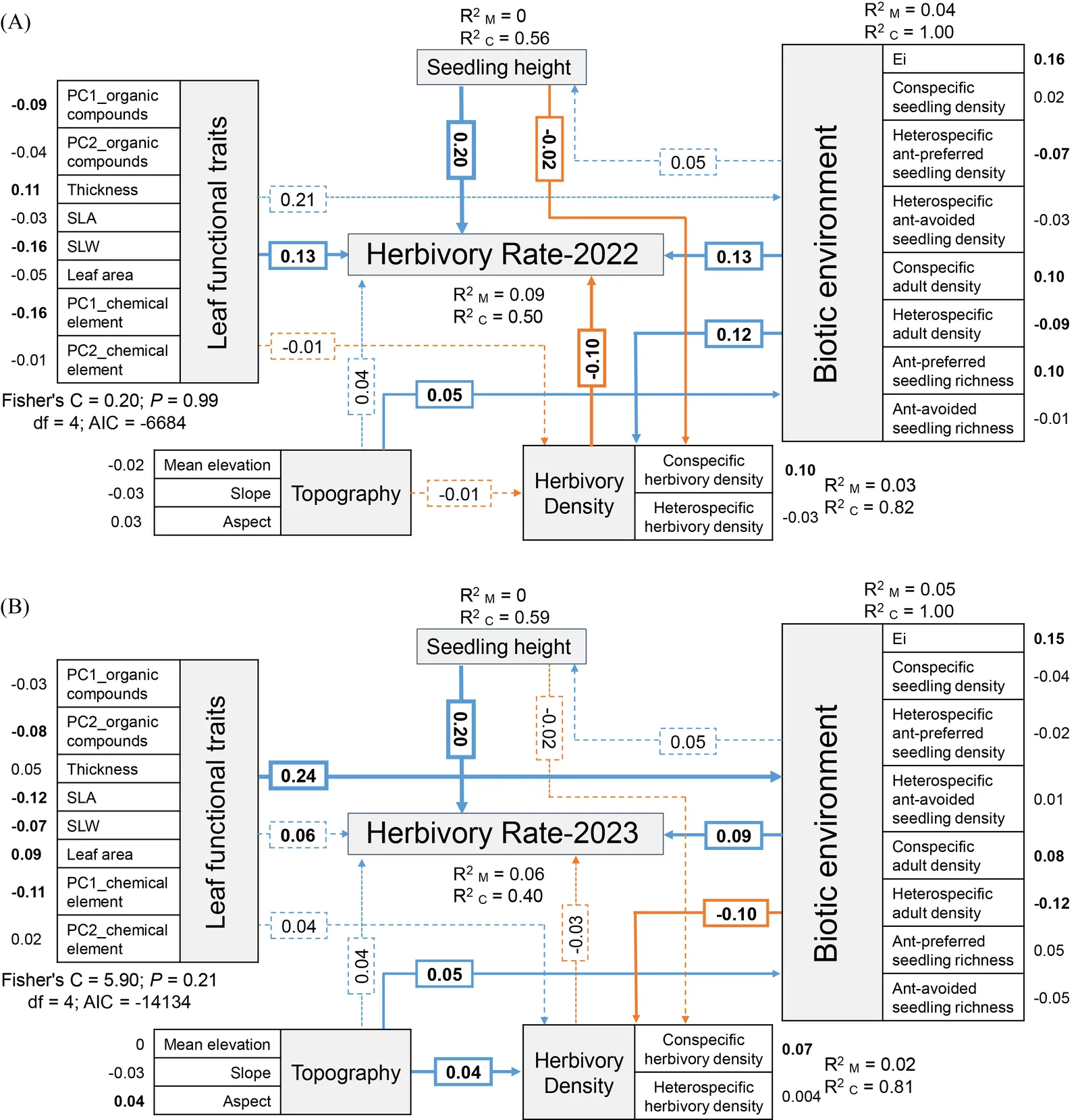
Direct and indirect divers of herbivory rate. PiecewiseSEM showing the direct and indirect effects of leaf functional traits, biotic environment, and seedling height on herbivory rate in 2022 (A, *n* = 2123) and 2023 (B, *n* = 2052). The Ei represents the strength of ant-plant interaction. The SLA is specific leaf area and the SLW is specific leaf weight. Conditional (C) and marginal (M) R^2^ scores depict the percentage of explained variance by predictor variables with and without considering the random effect of tree species. Blue lines signify positive effects, while orange lines indicate negative effect. The width of the lines is proportional to the coefficient values; solid lines signify positive relationships, whereas dashed lines signify negative ones. Numbers in bold represent statistically significant effects. Significance levels were set at ∗*P* < 0.05, ∗∗*P* < 0.01, ∗∗∗*P* < 0.001.

SEMs indicated that both seedling height and the biotic environment were essential direct drivers of herbivory rate (Fig. 3A and B). Notably, seedling height was strongest driver consistent with plant apparency hypothesis according to results of SEM. Within the biotic environmental factors, across both years, conspecific adult density had a positive impact on herbivory rate, and heterospecific adult density had a negative impact. These results are consistent with the JC, herd protection, and associational resistance hypotheses. Moreover, *Ei* exerted a positive effect on herbivory rate in both years. Further, the leaf functional traits could directly drive the herbivory rate, they can also indirectly influence herbivory rate via changes to the biotic environment (Fig. 3B). Although the effect of herbivory density on herbivory rate was not statistically significant, increasing conspecific herbivory density did tend to increase herbivory on focal seedlings (Fig. 3A, B). Finally, topography could affect the biotic environment, thereby influencing the herbivory rate. Seedling height showed stronger direct effects on seedling herbivory than other trait groups (Appendix S1: Table S7). Additionally, leaf functional traits influenced herbivory primarily through indirect pathways. In terms of total effects, seedling height emerged as the dominant driver (Appendix S1: Table S7).

## 4 Discussion

We determined that the average herbivory rate within this ecosystem was 9.91% in 2022 and 9.50% in 2023, which is close to the global average herbivory rate observed in woody species (10.74%; Galmán et al., 2018). The 10% threshold that has been identified as ecologically significant for regulating seedling performance and forest community composition (Moles and Westoby, 2004; Hanley and Sykes, 2009). The near-10% herbivory rate observed in our study is not trivial: as shown by Hanley and Sykes (2009), this level of foliar damage is sufficient to shift competitive hierarchies among seedling species, ultimately shaping community composition.

Our investigation identified several factors that potentially influence the level of herbivory experienced by individual seedlings. Notably, seedling height, heterospecific adult density, conspecific adult density, conspecific herbivory density, the presence of ant-plant associations, as well as the physical and chemical attributes of leaves were found to be significant determinants affecting standing herbivory levels.

### 4.1 A hierarchical interactive framework for seedling herbivory

Topography acts as a contextual modulator, exerting bottom-up indirect effects on ecological processes (Muscarella et al., 2020). The results of SEM revealed that topography could set the “abiotic context” for biotic interactions. For example, on steeper slopes, reduced soil moisture and nutrients lower conspecific adult tree density, which in turn reduces herbivore attraction mediated by Janzen-Connell effects, ultimately leading to decreased herbivory pressure on seedlings.

Across the 2022–2023 study period, the results obtained from both SEM and linear regression models indicated positive correlations between taller seedlings and increased herbivore pressure (Fig. 2, Fig. 3). These findings were consistent with the plant apparency hypothesis and in line with the conclusion drawn by Martini et al. (2021), thus echoing the foundational principles established by early scholars in the field of plant–herbivore interactions (Feeny, 1976). Consequently, the observations and herbivore dynamics elucidated in this study may be more applicable to the understory ecosystem, where an increase in height can dramatically increase visibility to herbivore.

Unexpectedly, our investigation showed that seedling herbivory increased with the intensity of interactions between ants and seedlings (Fig. 2, Fig. 3). This interesting finding suggests that ants may not provide protection against herbivory for focal seedlings. A potential underlying mechanism is that most plant species in the BDGS FDP do not produce EFNs, and instead attract predatory ants primarily when subjected to herbivore attack—thus forming an indirect association with ants via the herbivores themselves. However, we did observe that a higher density of heterospecific ant-associated seedlings generally reduced herbivory rate on focal seedlings, indicating that ants may indirectly protect plants located near ant-associated seedlings (Fig. 2, Fig. 3). This is consistent with recent research showing that non-EFN trees benefit from lower herbivore pressure due to the spillover of ants from neighboring ant-associated plants (Staab et al., 2023).

Our findings do support the Janzen-Connell hypothesis, in that higher conspecific adult density increased herbivory rate (Fig. 2, Fig. 3). This observation is consistent with previous studies indicating that a higher density of conspecifics is associated with increased rates of leaf damage in tropical forests (Downey et al., 2018). Our results suggest that conspecific adult density exhibit significant negative effects on seedling herbivory (Fig. 2). However, we also find an increase in the density of herbivory events on nearby conspecific seedlings leads to an increase in focal seedling herbivory (Fig. 2, Fig. 3), which indicated that herbivory damage becomes more severe only when conspecific seedlings are attacked by herbivores.

Our results indicated that leaf thickness was a significant predictor of herbivory (Fig. 2, Fig. 3), demonstrating a negative relationship where thicker leaves were less susceptible to consumption. This suggested that investing in physical defenses, such as thicker leaves, was crucial for mitigating herbivory pressure (Massad, 2023). Specific Leaf Weight (SLW), a proxy for leaf mechanical hardness (De Queiroz et al., 2013), exhibited a significant negative effect on seedling herbivory (Fig. 2, Fig. 3), indicating that seedlings with harder leaves (higher SLW) experienced lower herbivory damage (Coley and Barone, 1996; Massad et al., 2011). Unexcepted, we also found SLA had negative effect on herbivory rate (Fig. 2, Fig. 3), not consistent with previous researches (Wigley et al., 2014; Bouchenak-Khelladi et al., 2020), which indicated that herbivores preferred to medium-textured leaves.

Similarly, we also found that plant chemical content and the synthesis of secondary metabolites could strongly influence herbivory rate (Fig. 2, Fig. 3). This implies that investment in chemical defense may affect herbivory rate. In this analysis, we found physical leaf traits may exert a stronger control on herbivory rate, indicating that physical defense is more effective than chemical defense in this setting. There must be a trade-off between physical and chemical defense investment by plants (Eichenberg et al., 2015). Plants can effectively adjust their defense strategies by recognizing herbivores and investing in the most appropriate defensive traits accordingly (Fernández de Bobadilla et al., 2022). For example, chemical defenses are a response to specialist herbivores and are often induced, whereas physical defenses represent more generalist, constitutive defensive strategies (Lankau, 2007; Ali and Agrawal, 2012). Our results may imply a higher proportion of generalist herbivores in the BDGS plot, given that physical defense has a stronger effect on herbivory rate than chemical defense.

### 4.2 Herbivory-mediated drivers of seedling community assembly

Seedling establishment is widely regarded as a critical demographic bottleneck in plant life histories, characterized by the highest mortality rates across developmental stages (Hanley et al., 2004). Herbivory during this vulnerable phase acts as a powerful ecological filter, profoundly reshaping community structure and influencing trajectories of species coexistence (Barton and Hanley, 2013). Our results showed that taller seedling experienced higher herbivory (Fig. 2, Fig. 3), which suggests that herbivory could weaken the competitive ability of fast-growing species, thereby contributing to the coexistence of different species. Furthermore, our results indicated that conspecific herbivory density had positive effect on herbivory of focal seedling (Fig. 2, Fig. 3), confirmed the predictions of Janzen-Connell hypothesis (Comita et al., 2014), which means herbivory could reduce survival rate of the common species and then prevent rare species from being competitive exclusion.

Conversely, if herbivore density is too high, it can decimate the seedling bank, leading to recruitment failure and a simplification of community structure (Opedal et al., 2021). In our study, the mean foliar herbivory rate was 9.91% in 2022 and 9.50% in 2023, values that align closely with the global average reported by Galmán et al. (2018). This moderate level of herbivory suggests that it may serve as a stabilizing factor rather than a severe stressor, potentially promoting species coexistence within the subtropical forest.

### 4.3 Limitations and future directions

While our hierarchical framework explains key drivers of herbivory, the low model R^2^ highlights those additional unmeasured factors (e.g., soil nutrients, light, predator abundance) contribute to herbivory variation, emphasizing the complexity of plant–herbivore interactions in subtropical forests. Complete exclusion of historical leaf damage effects is impractical in long-term field surveys of evergreen species, as documented in comparable studies (e.g. Gherlenda et al., 2016; Jing et al., 2016). Future research targeting multi-year herbivory could incorporate deciduous species or destructive sampling (where feasible) to further reduce this potential bias.

While we considered topographic effects on soil moisture/nutrients indirectly, we did not directly measure soil properties (e.g., total nitrogen, phosphorus, organic matter content). Soil nutrients directly regulate leaf nutrient stoichiometry and defensive trait expression (e.g., high soil N increases leaf N content, attracting herbivores; low soil P enhances tannin synthesis, reducing herbivory; Ordoñez et al., 2009). Integrating direct soil nutrient measurements would clarify how abiotic bottom-up effects cascade through plant traits to modulate herbivory.

Light availability (e.g., canopy openness) was not quantified in our study, yet it strongly affects seedling apparency (via shade-induced height growth) and trait allocation (e.g., shade-tolerant seedlings invest more in defenses vs. light-capturing traits). Variation in canopy openness within and across plots could explain unaccounted herbivory heterogeneity—for example, high light increases seedling height (amplifying apparency effects) but may also enhance defensive trait expression, creating countervailing effects on herbivory.

We did not measure the abundance of herbivore predators (e.g., birds, spiders, beetles that predate on folivores), which exert strong top-down control on herbivore populations (Schmitz et al., 2010). In subtropical forests, predator diversity often correlates with microhabitat complexity (e.g., understory vegetation cover), and their absence from our model likely omits a key regulatory layer: for instance, high predator abundance could suppress herbivore populations even in high-apparency, high-conspecific density plots.

To address these gaps, future studies should: (1) Expand the SEM framework to include direct measurements of soil nutrients (e.g., ion chromatography for N/P), light availability (e.g., hemispherical photography for canopy openness), and predator abundance (e.g., pitfall traps, bird point counts); (2) Add paths linking these variables to herbivory (e.g., soil N → leaf N → herbivory; canopy openness → seedling height → herbivory; predator abundance → herbivory); (3) Adopt a multi-scale sampling design (plot-level + microhabitat-level) to capture fine-scale variation in these unmeasured factors. Integrating these variables will likely improve model R^2^ and provide a more comprehensive understanding of the full suite of drivers shaping seedling herbivory.

## CRediT authorship contribution statement

**Gang Zhou:** Writing-original draft, Writing-review and editing, Investigation, Visualization, Methodology, Formal analysis, Conceptualization. **Daniel F. Petticord:** Writing-review and editing, Methodology, Conceptualization. **Yao-Zhan Xu:** Investigation, Conceptualization. **Yuan-Zhi Qin:** Investigation, Conceptualization. **Biao Dong:** Investigation, Software, Conceptualization. **Xiu-Juan Qiao:** Writing-original draft, Writing-review and editing, Project administration, Funding acquisition, Investigation, Methodology, Formal analysis, Conceptualization. **Ming-Xi Jiang:** Writing-review and editing, Project administration, Funding acquisition, Investigation, Conceptualization.

## Data availability

We have delivered the R code and data to Zenodo. The code and data will also be permanently archived if the paper is accepted for publication. Data and code are available in Zenodo at https://doi.org/10.5281/zenodo.14130378.

## Declaration of competing interest

The authors declare that they have no known competing financial interests or personal relationships that could have appeared to influence the work reported in this paper.
