# Supplementary material for: Determinants of herbivory on seedlings in a subtropical forest: Insights from plant apparency, Janzen-Connell effects, and functional traits
Source: Plant Divers. 2026 Mar 17;48(5):927–35. doi: 10.1016/j.pld.2026.03.009 (PMC13615788; doi:10.1016/j.pld.2026.03.009)
Supplement: Supplementary file 1 [file mmc1.docx]

**Supplementary Materials**

**Determinants** **of herbivory on seedlings** **in a subtropical forest: Insights from plant apparency, Janzen-Connell effects, and** **functional traits**

**Gang Zhou, Daniel F. Petticord, Yao-Zhan Xu, Yuan-Zhi Qin, Biao Dong, Ming-Xi Jiang, Xiu-Juan Qiao^*^**

*Corresponding author. E-mail: qiaoxj@ccnu.edu.cn

Journal: *Plant Diversity*

**The standardized protocol for collecting and measuring plant chemical traits**

Sample Collection and Preparation: fully expanded leaves were collected from each seedling in June 2022, with 10 healthy individuals selected per species to ensure representativeness. Fresh weight of the leaves was measured immediately after collection, followed by high-resolution scanning to record leaf area (for subsequent trait calculations, e.g., specific leaf area). The leaves were then oven-dried at 72℃ for 48 hours to constant weight, and dry weight was recorded. Finally, the dried leaves were ground into a fine powder using a ball mill to pass through a 100-mesh sieve, which was stored in sealed centrifuge tubes at room temperature for subsequent biochemical or elemental analyses.

Digestion Procedure: approximately 0.25 g of the sample was weighed into a digestion tube. Then, 5 mL of concentrated sulfuric acid (H2SO4) and 1 mL of hydrogen peroxide (H₂O₂) were added. Next, we conducted the digestion process using a digestion furnace. We initially raised the temperature from ambient to 130℃ over a span of 10 minutes and kept it at that level for 8 minutes. We then further increased the temperature from 130℃ to 180℃ in just 2 minutes, maintaining it for 30 minutes. Finally, in a fume hood, we evaporated the acid at 180℃ for a total of 1 hour.

Sample Dilution: after digestion, the clear liquid was transferred to a 100 mL volumetric flask and then constant volume, and a 15 mL aliquot was pipetted into a centrifuge tube as the test extract solution.

Elemental Analysis: Initially, we fabricated standard solutions for individual elements and subsequently constructed a standard curve. Utilizing this curve as a reference, we then employed an elemental analyzer to automatically ascertain the concentrations of each sample.

Specially, approximately 0.25 g of the sample was weighed into a digestion tube. Then, 5 mL of concentrated sulfuric acid and 1 mL of hydrogen peroxide (H₂O₂) were added for digestion. After digestion, the clear liquid was transferred to a 100 mL volumetric flask, and a 15 mL aliquot was pipetted into a centrifuge tube as the test extract solution. Finally, the contents of chemical elements were determined Optical Emission Spectrometer.

The C and N determined by Elementar (FXL 950, America). Specifically, approximately 20 mg of the sample was weighed, encapsulated in a tin capsule, and introduced into Elementar for data acquisition. Other elements determined by Optical Emission Spectrometer (OPTIMA 8000DV, America).

The chlorophyll determined by SPAD (spad-502, Japan) and other organic compounds determined by assay kits. For example, approximately 0.1 g of the sample was weighed and homogenized with 1 mL of extraction solution in an ice bath. The homogenate was then centrifuged at 4℃ and 1000 rpm for 10 minutes. The resulting supernatant was collected as the test solution. Subsequently, the visible spectrophotometer was preheated for 30 minutes and calibrated to a wavelength of 562 nm, with zero adjustment performed using distilled water. The soluble protein content of the sample was calculated based on the absorbance of the test solution measured at 562 nm against a standard curve.

Fig. S1. The correlation among each continuous factor. Ei: the strength of interaction between ants and plants; S.con: conspecific seedling density; S.het_Y: heterospecific seedling density of ant preference; S.het_N: heterospecific seedling density of ant avoidance; A.con: conspecific adult density; A.het: heterospecific adult density; Diversity_Y: the species richness of ant preference; Diversity_N: the species richness of ant avoidance. Blue represents positive correlation, and red represents negative correlation. Significance levels: **P* < 0.05, ***P* < 0.01, ****P* < 0.001.

Fig. S2. The principal component analysis (PCA) of chemical elements ( C, N, P, K, Ca, Na, Mg, Fe, Al, Cu and Mn) and chemometrics (C:N, C:P, N:P). PC1 reflected nutrient availability (high N/P, low C:N/C:P), while PC2 reflected cation concentrations (Ca/Al).

Fig. S3. The principal component analysis (PCA) of organic matters including flavonoid, soluble protein, tannin, fructose, alkaloid and chlorophyll. The chlorophyll determined by SPAD (spad-502, Japan) and other organic compounds determined by assay kits. PC1 reflected defensive trait investment (high tannins/flavonoid), while PC2 reflected photosynthetic capacity (high chlorophyll).

Fig. S4. The conceptual diagram of structural equation model.

Table S1 The index of each category for leaf functional traits of 51 species.

| Function traits | Mean (95%CI) | Max | Min | Standard deviation | |
| --- | --- | --- | --- | --- | --- |
| **Leaf physical property** | | | | |  |
| Leaf thickness | 0.19(0.18,0.21) | 0.31 | 0.10 | 0.05 | |
| Specific leaf area (SLA) | 0.62(0.33,0.90) | 5.96 | 0.04 | 1.05 | |
| Specific leaf weight (SLW) | 6.59(4.73,8.45) | 37.58 | 0.23 | 6.78 | |
| Leaf area (LA) | 0.29(0.21,0.37) | 1.87 | 0.02 | 0.30 | |
| **Leaf chemical elements and chemometrics** | | | | |  |
| Potassium (K) | 8.66(6.67,10.65) | 34.50 | 0.76 | 7.25 | |
| Calcium (Ca) | 9.37(7.71,11.03) | 32.89 | 2.65 | 6.05 | |
| Iron (Fe) | 0.05(0.04,0.07) | 0.17 | -0.01 | 0.04 | |
| Aluminum (Al) | 3.04(0.95,5.14) | 31.33 | -0.01 | 7.63 | |
| Copper (Cu) | 0.01(0,0.01) | 0.02 | 0.00 | 0.00 | |
| Manganese (Mn) | 0.64(0.48,0.80) | 2.60 | -0.03 | 0.57 | |
| Sodium (Na) | 0.48(0.44,0.51) | 0.77 | 0.23 | 0.14 | |
| Magnesium (Mg) | 3.36(2.98,3.74) | 8.87 | 1.17 | 1.39 | |
| Phosphorus (P) | 1.16(1.07,1.25) | 2.20 | 0.65 | 0.33 | |
| Nitrogen (N) | 1.86(1.74,1.98) | 3.25 | 1.07 | 0.44 | |
| Carbon (C) | 45.03(44.20,45.87) | 50.00 | 36.40 | 3.05 | |
| Carbon/phosphorus | 41.64(38.48,44.79) | 71.74 | 21.59 | 11.50 | |
| Carbon/nitrogen | 25.60(23.88,27.32) | 41.03 | 13.59 | 6.27 | |
| Nitrogen/phosphorus | 1.65(1.55,1.75) | 2.53 | 1.04 | 0.35 | |
| **Leaf organic compounds** | | | | |  |
| Flavonoid | 33.86(28.01,39.71) | 80.57 | 5.04 | 21.31 | |
| Soluble protein | 128.48(102.38,154.58) | 483.08 | 15.06 | 95.10 | |
| Tannin | 6.05(5.80,6.30) | 7.47 | 3.15 | 0.91 | |
| Fructose | 14.02(11.95,16.10) | 50.32 | 3.96 | 7.55 | |
| Alkaloid | 3.18(2.42,3.93) | 15.25 | 0.82 | 2.76 | |
| Chlorophyll | 38.85(37.19,40.50) | 49.29 | 25.26 | 6.02 | |

Table S2 The herbivory rate of each species in Badagongshan FDP.

| Scientific Name | Family | Mean of herbivory-2022 | Mean of herbivory-2023 | Ei |
| --- | --- | --- | --- | --- |
| *Acer davidii* | Aceraceae | 0.049 | 0.035 | -0.637 |
| *Acer sinense* | Aceraceae | 0.136 | 0.113 | -0.039 |
| *Ardisia crenata* | Myrsinaceae | 0.085 | 0.076 | -0.763 |
| *Aucuba chinensis* | Cornaceae | 0.037 | 0.054 | -0.010 |
| *Callicarpa giraldii* | Verbenaceae | 0.100 | 0.163 | -0.297 |
| *Camellia costei* | Theaceae | 0.040 | 0.040 | -0.232 |
| *Camellia cuspidata* | Theaceae | 0.071 | 0.053 | 0.101 |
| *Camellia pitardii* | Theaceae | 0.153 | 0.110 | 0.312 |
| *Carpinus viminea* | Betulaceae | 0.089 | 0.096 | 0.440 |
| *Cornus elliptica* | Cornaceae | 0.095 | 0.062 | -0.152 |
| *Daphniphyllum macropodum* | Daphniphyllaceae | 0.086 | 0.070 | 0.575 |
| *Dendropanax dentigerus* | Araliaceae | 0.053 | 0.057 | 0.337 |
| *Enkianthus serrulatus* | Ericaceae | 0.114 | 0.111 | -0.569 |
| *Euonymus myrianthus* | Celastraceae | 0.070 | 0.073 | -0.260 |
| *Eurya brevistyla* | Theaceae | 0.116 | 0.095 | 0.284 |
| *Euscaphis japonica* | Staphyleaceae | 0.043 | 0.022 | -0.400 |
| *Fagus lucida* | Fagaceae | 0.083 | 0.157 | 0.312 |
| *Ficus deltoidea* | Moraceae | 0.057 | 0.093 | -0.555 |
| *Gamblea ciliata* | Araliaceae | 0.028 | 0.028 | 0.533 |
| *Hydrangea strigosa* | Saxifragaceae | 0.131 | 0.235 | -0.272 |
| *Ilex pernyi* | Aquifoliaceae | 0.025 | 0.028 | -1.000 |
| *Ilex suaveolens* | Aquifoliaceae | 0.118 | 0.109 | -0.010 |
| *Ilex szechwanensis* | Aquifoliaceae | 0.055 | 0.062 | -0.342 |
| *Lindera pulcherrima* | Lauraceae | 0.097 | 0.086 | -0.014 |
| *Litsea elongata* | Lauraceae | 0.102 | 0.083 | -0.215 |
| *Machilus pauhoi* | Lauraceae | 0.105 | 0.090 | 0.084 |
| *Meliosma oldhamii* | Sabiaceae | 0.067 | 0.129 | -0.186 |
| *Photinia amphidoxa* | Rosaceae | 0.112 | 0.156 | 0.637 |
| *Photinia beauverdiana* | Rosaceae | 0.184 | 0.092 | 0.357 |
| *Photinia davidiana* | Rosaceae | 0.059 | 0.042 | -0.010 |
| *Pittosporum glabratum* | Pittosporaceae | 0.025 | 0.019 | -0.664 |
| *Pittosporum trigonocarpum* | Pittosporaceae | 0.035 | 0.033 | -0.453 |
| *Prunus grayana* | Rosaceae | 0.117 | 0.083 | -0.637 |
| *Quercus engleriana* | Fagaceae | 0.272 | 0.199 | -0.489 |
| *Quercus multinervis* | Fagaceae | 0.269 | 0.264 | 0.031 |
| *Quercus shennongii* | Fagaceae | 0.165 | 0.148 | 0.412 |
| *Rhododendron fortunei* | Ericaceae | 0.087 | 0.099 | -0.489 |
| *Rhododendron mariesii* | Ericaceae | 0.017 | 0.063 | -0.707 |
| *Schima parviflora* | Theaceae | 0.128 | 0.125 | 0.219 |
| *Skimmia reevesiana* | Rutaceae | 0.045 | 0.028 | 0.466 |
| *Sorbus folgneri* | Rosaceae | 0.054 | 0.099 | -0.400 |
| *Symplocos anomala* | Symplocaceae | 0.103 | 0.082 | -0.208 |
| *Symplocos groffii* | Symplocaceae | 0.074 | 0.057 | 0.116 |
| *Symplocos macrophylla* | Symplocaceae | 0.122 | 0.105 | 0.212 |
| *Symplocos pseudobarberina* | Symplocaceae | 0.083 | 0.079 | 0.007 |
| *Symplocos sumuntia* | Symplocaceae | 0.088 | 0.067 | -0.549 |
| *Symplocos tanakana* | Symplocaceae | 0.130 | 0.148 | 0.522 |
| *Ternstroemia gymnanthera* | Theaceae | 0.012 | 0.054 | 0.015 |
| *Vaccinium bracteatum* | Ericaceae | 0.078 | 0.064 | -0.076 |
| *Viburnum erosum* | Caprifoliaceae | 0.129 | 0.126 | 0.330 |
| *Viburnum setigerum* | Caprifoliaceae | 0.073 | 0.148 | -0.535 |

Table S3 The coefficients of herbivory rate models about biotic environment factors.

|  | **Estimate** | **SE** | **t value** | **Pr(>\|t\|)** |
| --- | --- | --- | --- | --- |
| **Herbivory rate of 2022 (n = 2123)** | | | | |
| (Intercept) | 0.467 | 0.017 | 27.694 | **<0.0001** |
| E_i_ | 0.082 | 0.012 | 6.807 | **<0.0001** |
| Conspecific seedling density | 0.001 | 0.002 | 0.818 | 0.413 |
| Heterospecific ant-preferred seedling density | -0.004 | 0.002 | -2.224 | **0.026** |
| Heterospecific ant-avoided seedling density | -0.002 | 0.002 | -0.832 | 0.405 |
| Conspecific adult density | 0.573 | 0.125 | 4.582 | **<0.0001** |
| Heterospecific adult density | -0.122 | 0.031 | -3.915 | **<0.0001** |
| Ant-preferred seedling richness | 0.011 | 0.004 | 2.837 | **0.004** |
| Ant-avoided seedling richness | -0.001 | 0.004 | -0.341 | 0.733 |
| **Herbivory rate of 2023 (n = 2052)** | | | | |
| (Intercept) | 0.493 | 0.017 | 28.672 | **<0.0001** |
| E_i_ | 0.077 | 0.012 | 6.143 | **<0.0001** |
| Conspecific seedling density | -0.003 | 0.002 | -1.648 | 0.100 |
| Heterospecific ant-preferred seedling density | -0.001 | 0.002 | -0.443 | 0.656 |
| Heterospecific ant-avoided seedling density | 0 | 0.002 | 0.168 | 0.866 |
| Conspecific adult density | 0.501 | 0.136 | 3.673 | **<0.001** |
| Heterospecific adult density | -0.159 | 0.031 | -5.089 | **<0.0001** |
| Ant-preferred seedling richness | 0.006 | 0.004 | 1.406 | 0.160 |
| Ant-avoided seedling richness | -0.005 | 0.004 | -1.378 | 0.168 |

Table S4 The coefficients of herbivory rate models about leaf functional traits.

|  | **Estimate** | **SE** | **t value** | **Pr(>\|t\|)** |
| --- | --- | --- | --- | --- |
| **Herbivory rate of 2022 (n = 2123)** | | | | |
| (Intercept) | 0.340 | 0.021 | 15.920 | **<0.0001** |
| PC1_organic compounds | -0.123 | 0.032 | -3.880 | **0.0001** |
| PC2_organic compounds | -0.065 | 0.043 | -1.523 | 0.128 |
| Thickness | 0.465 | 0.110 | 4.221 | **<0.0001** |
| SLA | -0.008 | 0.010 | -0.743 | 0.457 |
| SLW | -0.005 | 0.001 | -6.444 | **<0.0001** |
| LA | -0.048 | 0.040 | -1.224 | 0.221 |
| PC1_chemical compounds | -0.430 | 0.064 | -6.677 | **<0.0001** |
| PC2_chemical compounds | -0.012 | 0.029 | -0.426 | 0.700 |
| **Herbivory rate of 2023 (n = 2052)** | | | | |
| (Intercept) | 0.350 | 0.022 | 16.251 | **<0.0001** |
| PC1_organic compounds | -0.037 | 0.033 | -1.116 | 0.264 |
| PC2_organic compounds | -0.142 | 0.045 | -3.140 | **0.002** |
| Thickness | 0.204 | 0.109 | 1.869 | 0.062 |
| SLA | -0.031 | 0.010 | -2.926 | **0.003** |
| SLW | -0.002 | 0.001 | -2.814 | **0.005** |
| LA | 0.098 | 0.044 | 2.228 | **0.026** |
| PC1_chemical compounds | -0.305 | 0.068 | -4.507 | **<0.0001** |
| PC2_chemical compounds | 0.022 | 0.031 | 0.708 | 0.479 |

Table S5 The coefficients of herbivory rate models about herbivory density factors.

|  | **Estimate** | **SE** | **t value** | **Pr(>\|t\|)** |
| --- | --- | --- | --- | --- |
| **Herbivory rate of 2022 (n = 2123)** | | | | |
| (Intercept) | 0.400 | 0.007 | 59.212 | **<0.0001** |
| Conspecific herbivory density | 0.066 | 0.015 | 4.527 | **<0.0001** |
| Heterospecific herbivory density | -0.010 | 0.007 | -1.397 | 0.162 |
| **Herbivory rate of 2023 (n = 2052)** | | | | |
| (Intercept) | 0.385 | 0.007 | 58.066 | **<0.0001** |
| Conspecific herbivory density | 0.063 | 0.018 | 3.430 | **<0.001** |
| Heterospecific herbivory density | 0.001 | 0.007 | 0.177 | 0.860 |

Table S6 The coefficients of herbivory rate models about topography.

|  | **Estimate** | **SE** | **t value** | **Pr(>\|t\|)** |
| --- | --- | --- | --- | --- |
| **Herbivory rate of 2022 (n = 2123)** | | | | |
| (Intercept) | 0.763 | 1.049 | 0.728 | 0.467 |
| Mean elevation | -2.514×10^-4^ | 7.327×10^-4^ | -0.338 | 0.735 |
| Slope | -3.770×10^-4^ | 5.889×10^-4^ | -0.640 | 0.522 |
| Aspect | 5.497×10^-5^ | 3.891×10^-5^ | 1.413 | 0.158 |
| **Herbivory rate of 2023 (n = 2052)** | | | | |
| (Intercept) | 0.393 | 1.075 | 0.366 | 0.715 |
| Mean elevation | -1.083×10^-6^ | 7.613×10^-4^ | -0.001 | 0.999 |
| Slope | -4.260×10^-4^ | 6.113×10^-4^ | -0.697 | 0.486 |
| Aspect | 7.893×10^-5^ | 3.972×10^-5^ | 1.987 | **0.047** |

Table S7 The direct and indirect effects of each group on herbivory rate.

|  | **Direct effect** | **Indirect effect** | **Total effect** |
| --- | --- | --- | --- |
| **SEM of 2022 (n = 2123)** | | | |
| Seedling Height | 0.200 | 0.002 | 0.202 |
| Leaf functional traits | 0.130 | 0.03 | 0.133 |
| Biotic environment | 0.130 | -0.002 | 0.128 |
| Herbivory Density | -0.100 | 0 | -0.100 |
| Topography | 0.040 | 0.008 | 0.048 |
| **SEM of 2023 (n = 2052)** | | | |
| Seedling Height | 0.200 | 0.001 | 0.201 |
| Leaf functional traits | 0.060 | 0.021 | 0.081 |
| Biotic environment | 0.090 | 0.013 | 0.103 |
| Herbivory Density | -0.030 | 0 | -0.030 |
| Topography | 0.04 | 0.003 | 0.043 |
